# Food intake biomarkers for apple, pear, and stone fruit

**DOI:** 10.1186/s12263-018-0620-8

**Published:** 2018-11-29

**Authors:** Marynka Ulaszewska, Natalia Vázquez-Manjarrez, Mar Garcia-Aloy, Rafael Llorach, Fulvio Mattivi, Lars O. Dragsted, Giulia Praticò, Claudine Manach

**Affiliations:** 1 Research and Innovation Centre Food Quality and Nutrition, Fondazione Edmund Mach, Via Mach 1, San Michele all’Adige, 38010 Trento, Italy; 2 Human Nutrition Unit, Université Clermont Auvergne, INRA, F63000 Clermont-Ferrand, France; 3 Department of Nutrition, Exercise and Sports, University of Copenhagen, Copenhagen, Denmark; 4 Biomarkers and Nutrimetabolomic Laboratory, Department of Nutrition, Food Sciences and Gastronomy, XaRTA, INSA, Faculty of Pharmacy and Food Sciences, Campus Torribera, University of Barcelona, Barcelona, Spain; 5 CIBER de Fragilidad y Envejecimiento Saludable (CIBERFES), Instituto de Salud Carlos III, Barcelona, Spain; 6 Center Agriculture Food Environment, University of Trento, San Michele all’Adige, Trento, Italy

**Keywords:** Apple, Pear, Quince, Pome fruit, Stone fruit, Cherry, Plum, Prune, Apricot, Peach, Nectarine, Biomarkers, Intake

## Abstract

Fruit is a key component of a healthy diet. However, it is still not clear whether some classes of fruit may be more beneficial than others and whether all individuals whatever their age, gender, health status, genotype, or gut microbiota composition respond in the same way to fruit consumption. Such questions require further observational and intervention studies in which the intake of a specific fruit can be precisely assessed at the population and individual levels. Within the Food Biomarker Alliance Project (FoodBAll Project) under the Joint Programming Initiative “A Healthy Diet for a Healthy Life”, an ambitious action was undertaken aiming at reviewing existent literature in a systematic way to identify validated and promising biomarkers of intake for all major food groups, including fruits. This paper belongs to a series of reviews following the same BFIRev protocol and is focusing on biomarkers of pome and stone fruit intake. Selected candidate biomarkers extracted from the literature search went through a validation process specifically developed for food intake biomarkers.

## Background

### Introduction

Fruit is an essential component of a healthy diet. In a comparative risk assessment of global disease burden attributable to 67 risk factors, diets low in fruit were estimated to account for 30% of ischemic heart disease disability-adjusted life years worldwide and ranked among the five leading risk factors and as the first dietary factor for global disease burden and mortality [1]. Large prospective cohort studies, increasingly supported by well-designed randomized clinical trials, have conclusively established the protective effects of high fruit intake regarding hypertension, cardiovascular disease, and stroke, with some evidence of a dose-response relationship [2–5]. High intake of fruit and vegetables (F&V) have also been associated with prevention of other chronic diseases such as several cancer types, obesity and type 2 diabetes, or neurodegenerative diseases, however, mixed results were reported, and the overall evidence is more limited [6–9]. There is a strong need for more research in the field to answer important pending questions and guide the development of more efficient public health policies and healthy food production. One major interrogation is whether the total quantity of fruit consumed is the most important factor or whether the intake of particular fruits or groups of fruit, or a high diversity, matters. Some fruits expected to provide important amount of specific bioactive compounds in the human diet, such as pomegranate, orange, or cranberry, have received much interest in the last decades. Due to the diversity of fruit composition regarding bioactives, it is important to evaluate the specific health effects of the individual fruits, to identify their protective constituents and biological targets and eventually to determine the most beneficial associations. This is particularly relevant for the cancer-protective or the anti-obesity effects of fruit, which were observed to differ for various types of fruit [6, 10]. Another question concerns the inter-individual variability in response to fruit consumption. It is not clear whether everyone, regardless of age, gender, lifestyle, gut microbiota composition, or genotype, responds similarly to fruit consumption and if there is a risk associated with high fruit intake for some individuals. All these questions require further investigations in human intervention and large prospective studies in which an accurate assessment of fruit intake can be made for every subject, not only of total intake of fruit but also for the intake of several classes of fruit and individual fruits.

Fruit intake is traditionally assessed with dietary questionnaires. The usual consumption of total F&V is sometimes the only information inquired in Food Frequency Questionnaires (FFQs), and even in the most detailed FFQs classes of fruit (such as pome, citrus, drupes, berries, nuts….) may be distinguished, but rarely individual species (apples, pears, oranges, grapefruits, etc.) and almost never down to the variety. Repeated 24-h recalls are more precise but still biased by self-reporting inaccuracy. The consumption of fruit and vegetables has been shown to be particularly prone to overestimation in dietary questionnaires, at least for a fraction of the population [11]. Complementary to questionnaires, biomarkers such as plasma vitamin C and plasma carotenoids have been widely used [5, 12]. However, as shown in a systematic review and meta-analysis of 19 intervention studies, these biomarkers can reflect group-level differences for assessing compliance to F&V interventions, but are not accurate enough to precisely reflect individual-level intakes [13]. These traditional biomarkers give heterogeneous responses depending on the type of F&V consumed and are affected by a range of intrinsic and environmental factors.

The Food Biomarker Alliance (FoodBAll), a project funded by the Joint Programming Initiative, “A Healthy Diet for a Healthy Life” (http://www.healthydietforhealthylife.eu/), has undertaken a systematic evaluation of traditional and newly discovered biomarkers of food intake (BFIs). Guidelines were established for conducting a systematic literature search dedicated to food intake biomarkers [14] and for evaluating their level of validation using a set of consensus criteria [15]. The guidelines were applied for more than 140 foods from all major food groups: fruit and vegetables, meats, fish and other marine foods, dairy products, cereals and wholegrains, alcoholic and non-alcoholic beverages, vegetable oils, nuts, and spices and herbs (http://foodmetabolome.org/wp3). The present article presents the results of the in-depth exploration of possible biomarkers of intake for important classes of fruit, the pome and stone fruit.

### Methods

#### Selection of food groups

The most widely consumed pome and stone fruit were inventoried [16]. For pome fruit, the apple (*Malus domestica* Borkh.) and pear (*Pyrus communis* L.) were selected, as well as quince (*Cydonia oblonga* Miller), which is less frequently consumed as jams, marmalade, jellies, or “pâte de fruit.” For stone fruit, sweet cherry (*Prunus avium* L.), sour cherry (*Prunus cerasus* L.), plum and prune (*Prunus domestica* L.), apricot (*Prunus armeniaca* L.), peach (*Prunus persica* (L.) Batsch), and nectarine (*Prunus persica var. nucipersica* (Borkh.) C.K.Schneid.) were covered. Botanical genus and generic fruit group names were also used in the search, as described below, to ensure that no other important pome or stone fruits were missed.

#### Search for relevant BFI research papers

An extensive literature search was carried out to collect all available information on the existing and new candidate BFIs for the selected fruits. The BFIRev protocol (Food Intake Biomarker Reviews) described previously was followed [14]. Briefly, a primary search was performed in three databases, Scopus, PubMed central, and Web of Science with the name of the specific fruit and its botanical genus, i.e., (pear OR pyrus*), (apple* OR *Malus domestica*), (quince OR *Cydonia oblonga*), and (plum OR peach OR nectarine OR cherry OR apricot OR prunus OR drupe* OR stone fruit) along with the common keywords: AND (urine OR plasma OR serum OR excretion OR blood) AND (human* OR men OR women OR patient* OR volunteer* OR participant*) AND (biomarker* OR marker* OR metabolite* OR biokinetics OR biotransformation OR pharmacokinetics OR bioavailability OR ADME) AND (intake OR meal OR diet OR ingestion OR administration OR consumption OR eating OR drink*). Keywords were used in the fields [Topic], [All fields], and [Article Title/Abstract/Keywords] for Web of Science, PubMed, and Scopus, respectively. All searches were carried out in March 2016 and updated in May 2017. Only papers in English language were considered eligible, and no restriction on the date of publication was applied. Articles showing results of human intervention studies (randomized controlled trials, acute, short-term or long-term studies) or observational studies (cohort, case-control, cross-sectional studies) were considered eligible. After duplicate removal, a first selection of papers was performed according to relevance of abstract and title. Full texts were obtained for the selected articles and further assessed for eligibility according to their relevance in determining BFIs for pome and stone fruit. Some of the publications found in the reference list of the selected articles were also included at this stage.

**Fig. 1:**
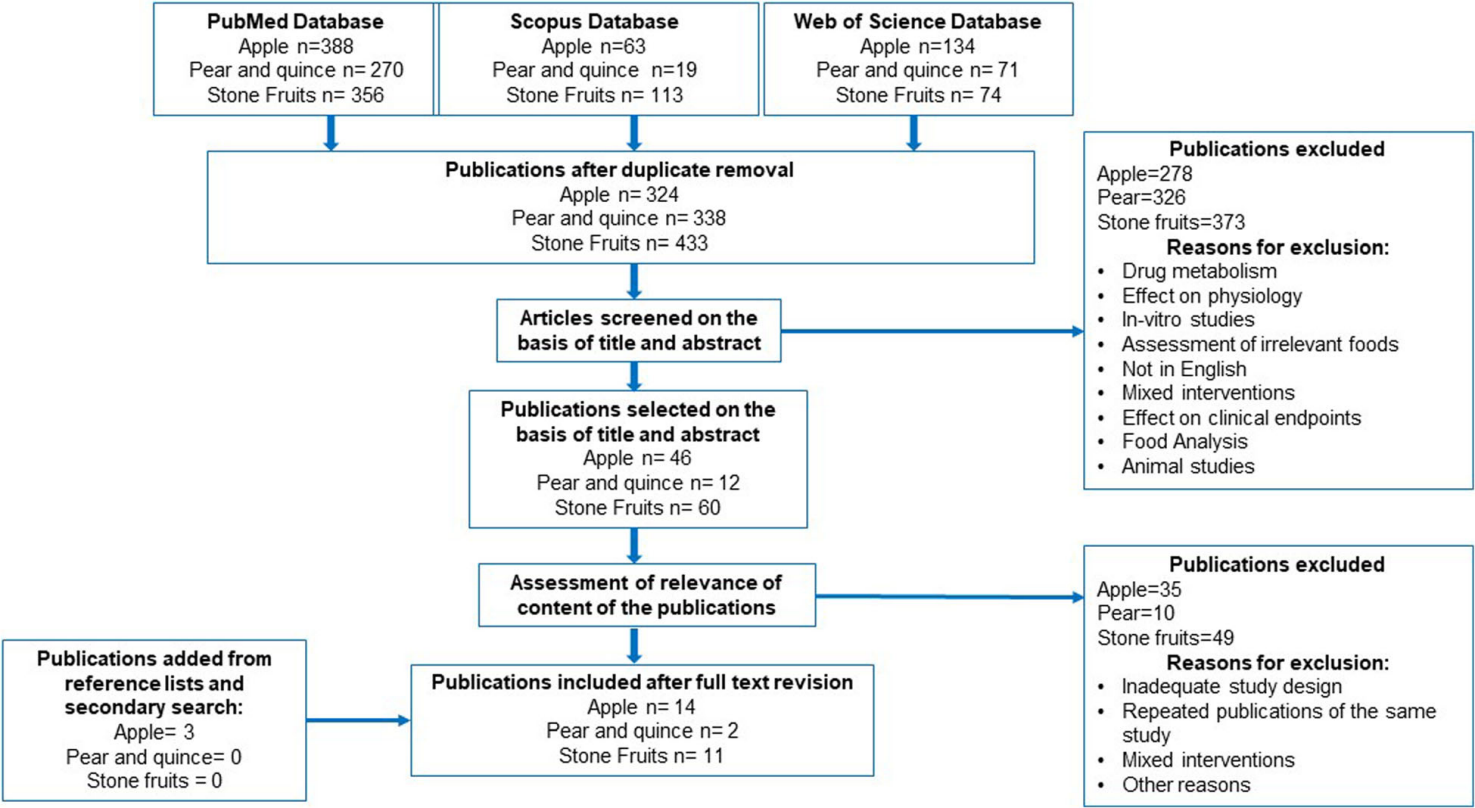
Flow diagram of study selection according to the BFIRev procedure

The process of selection of the articles identifying or using potential biomarkers of intake is outlined in Fig. 1.

#### Identification and characterization of candidate BFIs

For each potential biomarker identified, a secondary search allowed to retrieve relevant information to assess the quality of the individual biomarkers, regarding their specificity, their pharmacokinetics, dose-response relationship, the robustness, and reliability of their method of analysis, in order to qualify their use as BFIs according to the validating scheme established by Dragsted et al. [15].

The name of the potential biomarkers and their synonyms were queried in the previously mentioned databases along with AND (biomarker* OR marker* OR metabolite* OR biokinetics OR biotransformation OR pharmacokinetics OR bioavailability OR ADME). Additionally, the compounds were searched manually in the online databases, HMDB (https://www.hmdb.ca), FooDB (http://foodb.ca/), Phenol-Explorer (http://phenol-explorer.eu/), Dictionary of Food Compounds (http://dfc.chemnetbase.com/faces/chemical/ChemicalSearch.xhtml), Duke’s phytochemical and ethnobotanical databases (https://phytochem.nal.usda.gov/phytochem/search), eBASIS (http://ebasis.eurofir.org/Default.asp), Knapsack (http://kanaya.naist.jp/knapsack_jsp/top.html), and PhytoHub (http://phytohub.eu) to determine all the possible dietary or metabolic origins of the candidate BFIs.

Specific and non-specific biomarkers were discussed in the text, while only the most plausible candidate BFIs for pome fruit have been reported in Table 1 along with the information related to study designs and analytical methods. The non-retained compounds for pome fruit and stone fruit are listed in Tables 2 and 3, respectively, along with the main reasons for exclusion and with the corresponding references for an exhaustive presentation of the results. The tables have been reviewed and agreed upon by all authors and no additional markers were suggested.

**Table 1:** List of studies reporting candidate biomarkers for pome fruit consumption

Dietary factor	Study design	Number of subjects	Analytical method	Biofluid	Discriminating metabolites/candidate biomarkers	Primary reference(s)
Apple						
Apple (24-h recalls, 58.7 ± 113.5 g/day^+^)	Observational study	53 (31 women, 22 men)	HPLC-MS	Urine (spot and 24 h)	• Phloretin • Other non-specific polyphenol metabolites	[40]
Apple (FFQ and food diaries, 47 (3–140)g/day^*^)	Observational study	119 (all women)	HPLC-ESI-MS	Urine (24 h) Plasma	• Phloretin • Other non-specific compound	[42]
Apple (24-h recall, 61 (0–317) g/day^*^)	Observational study: 5 months of free access to fruit basket in working place.	79	HPLC-ESI-MS	Urine (24 h)	• Phloretin • Other polyphenols for other fruits	[41]
Apple and pear (24-h recalls, 228 ± 239 g/day^+^)	Observational study	481	Untargeted HPLC-TOF-MS	Urine (24 h)	• Phloretin glucuronide • Other non-specific epicatechin metabolites	[17]
40 g of lyophilized apples: polyphenol-rich vs polyphenol-poor apples	Double-blind, randomized cross-over trial, 4-week periods	30 (all men)	LC-MS	Morning spot urine	• Phloretin	[47]
25 g of unripe apple processed in powder	Randomized cross-over study (two 1-day interventions: (1) 50 g OGTT and (2) 50 g OGTT+ 25 g apple powder)	6 (all women)	LC-MS	Urine (0 h, 0-2 h, 2–4 h)	• Phloretin • Phloretin glucuronide	[45]
1 L cloudy apple juice	Kinetics intervention, single dose	11 (healthy ileostomy subjects)	HPLC-DAD; HPLC-ESI-MS/MS	Ileostomy fluid (0 h, 1 h, 2 h, 4 h, 6 h, and 8 h)	• Phloretin 2′-*O*-xyloglucoside • Phloretin 2′-*O*-glucuronide • Phloretin • Other non-specific polyphenol metabolites	[46]
0.7 L of apple smoothie	Single dose, kinetic study	10 (healthy ileostomy persons)	HPLC-DAD and HPLC-MS/MS	Ileostomy fluid (0 h, 1 h, 2 h, 4 h, 6 h, and 8 h)	• Phloretin 2′-*O*-xyloglucoside • Phloretin 2′-*O*-glucuronide (and isomers) • Phloretin • Other non-specific polyphenol metabolites	[95]
1 kg of apple (organic vs conventional)	Randomized, cross-over single-dose study (2 experimental days, 1 per intervention)	6 (all men)	HPLC-MS	Plasma (0 h, 1 h, 2 h, 3 h, 4 h, 5 h, 6 h, 9 h, 12 h, 24 h)	• Phloretin	[43]
500 g/day apple for 4 weeks (organic vs conventional vs no apple)	Double-blind, randomized parallel study (3 interventions: (1) organic apple, (2) conventional apple, (3) control)	43 (all men)	HPLC-MS	Plasma (day 0 and day 28 24 h after last intake of fruit)	• Phloretin	[43]
Apple (low: 200 ± 10 g, medium: 400 ± 10 g and high: 790 ± 10 g consumption)	Acute parallel study with three groups	30 (14 women, 16 men)	HPLC-ESI-QTrap	Urine (0 h, 0–2 h, 2–4 h, 4–6 h, 6–8 h, 8–10 h, 10–12 h, 12–14 h, 14–16 h, 48 h morning spot, 72 h morning spot, 96 h morning spot)	• Phloretin • Other non-specific polyphenol metabolites	[48]
Pear						
Pear (0.6 g/kg/h) vs Banana (0.6 g/kg/h) vs water (3 ml/kg/h)	Cross-over, randomized controlled trial (3 experimental days, 1 per intervention)	20 (all men)	Untargeted UPLC-QTOF-MS	Plasma (T0, immediately after cycling, 1.5 h after cycling trial and 21-h post exercise)	• Arbutin • Hydroquinone sulfate • Other non-specific compounds (sugars, polyphenol microbial metabolites)	[64]
Pear (1 fruit, as part of a high Hydroquinone diet)	Controlled Intervention study (3 experimental days, 1 per intervention: high hydroquinone meal with pear, low hydroquinone and acetaminophen)	4 (2 women, 2 men)	GC-ED	Plasma (30, 60, 120 min after meal) Urine (2-h interval collection in a 8-h period after meal)	• Conjugated hydroquinone	[65]

*Abbreviations*: *OGTT* oral glucose tolerance test, *HPLC-MS* high-performance liquid chromatography-mass spectrometry, *HPLC-ESI-MS* high-performance liquid chromatography-electron spray ionization-mass spectrometry, *HPLC-TOF-MS* high-performance liquid chromatography-time-of-flight-mass spectrometry, *LC-MS* liquid chromatography-mass spectrometry, *HPLC-DAD* high-performance liquid chromatography-with diode-array-detection, *UPLC-QTOF-MS* ultra-performance liquid chromatography-quadrupole-time-of-flight-mass spectrometry, *GC-ED* gas chromatography-electron capture detector

^+^Data reported in mean and SD

^*^Data reported in medians and percentiles

**Table 2:** List of non-retained compounds for pome fruit

Food item	Compound	PhytoHub ID	HMDB ID	Biofluid	Reasons for exclusion	References
Apple	Dihydroxyphenyl-γ-valerolactone, dihydroxyphenyl-γ-valerolactone sulfate	PHUB001060 -	HMDB0029185 -	Urine	Non-specific flavanol metabolite, common for many fruits and vegetables, too variable background	[17, 28]
Apple	D-(-)-quinic acid	PHUB000633	HMDB0003072	Ileostomy fluid, plasma, urine	Non-specific, common for many fruits,	[18, 40, 43, 46, 95]
Apple	p-Coumaric acid, hydroxycinnamic acid, methyl caffeic acid, methyl p-coumarate	PHUB000590 PHUB000588 - PHUB001887	HMDB0030677, - - -	Ileostomy fluid, plasma, urine	Non-specific, common for many fruits,	[18, 40, 43, 46, 95]
Apple	1-Caffeoylquinic acid, 3-caffeoylquinic acid, 4-caffeoylquinic acid, 5-caffeoylquinic acid, 3-p-coumaroylquinic acid, 4-p-coumaroylquinic acid, 5-p-coumaroylquinic acid,	PHUB000514 PHUB000530 PHUB000537 PHUB000585 PHUB000534 PHUB000545 PHUB000558	HMDB0030652, HMDB0003164 HMDB0040690, HMDB0030653, HMDB0029681, - -	Ileostomy fluid	Non-specific, common for many fruits and vegetables, lack in plasma and urine	[46, 95]
Apple	Catechin, epicatechin, (epi)catechin-*O*-sulfate, oligomeric procyanidins, procyanidin B2, procyanidin B5	PHUB000261 PHUB001241 PHUB001040 - PHUB000277 PHUB000280	HMDB0002780, HMDB0001871, HMDB0012467 - HMDB0033973, HMDB0038366	Ileostomy fluid, plasma, urine	Non-specific, common for many fruits	[17, 20, 21, 46, 95]
Apple	Quercetin, kaempferol, isorhamnetin, quercetin 3-*O*-arabinoside, quercetin 3-*O*-rhamnoside, quercetin 3-*O*-glucoside, quercetin 3-*O*-galactoside, quercetin 3-*O*-xyloside	PHUB000702 PHUB000672 PHUB000662 PHUB000654 PHUB001763 PHUB000661 PHUB001454 PHUB000716	HMDB0005794, HMDB0005801, HMDB0002655, HMDB0033795, HMDB0033751, HMDB0037362, HMDB0030775, HMDB0037927,	Ileostomy fluid, urine	Non-specific, common for many fruits. Glycosides expected only in ileostomy fluid	[40, 41, 46, 48, 95]
Apples and pears	Threitol	–	HMDB0004136	Plasma	Alcohol-sugar present in many fruit and fermented foods	[18]
Apples and pears	Indolepropionate	–	HMDB0002302	Plasma	Microbial metabolite of tryptophan	[18]
Apples and pears	3-Phenylpropionate	PHUB001078	HMDB0000764	Plasma	Non-specific microbial metabolite of polyphenols	[18]
Pear	Sugars: xylose, fructose, ribitol, mannitol, sorbitol, arabitol, xylitol	–	HMDB00098; HMDB00660; HMDB00508; HMDB00765; HMDB00247; HMDB00568; HMDB02917	Plasma	Non-specific sugars and alcohol sugars; found in many fruits and other foods	[64]
Pear	Eugenol sulfate	PHUB001888	–	Plasma	Metabolite of eugenol present in several fruits. Non-specific metabolite	[64]
Pear	2-Isopropylmalate	–	HMDB00402	Plasma	Non-specific, scarce information on kinetics and dose-response	[64]
Pear	Vanillic alcohol sulfate	–	–	Plasma	Metabolite of vanillic acid, which is found in fruit; scarce information on kinetics and dose-response	[64]
Pear	3-(4-Hydroxyphenyl) propionate; 4-hydroxyhippurate; hippuric acid	PHUB001177 PHUB001334 PHUB001174	HMDB02199; HMDB13678; HMDB00714	Plasma	Metabolites of phenolic acids and other polyphenols; scarce information on kinetics and dose-response	[64]
Pear	4-Allylphenol sulfate	PHUB001891	–	Plasma	Metabolite of 4-allyphenol found in essential oils and several fruits. Non-specific compound	[64]

*Abbreviations*: *HMDB ID* human metabolome database identification number, http://www.hmdb.ca; PhytoHub ID (http://phytohub.eu/)

**Table 3:** List of non-retained compounds for stone fruit

Food item	Compound	PhytoHub ID	HMDB ID	Biofluid	Reasons for exclusion	References
Cherry	Cyanidin 3-rutoside, cyanidin 3-*O*-glucoside, cyanidin-3-glucosylrutinoside	PHUB000504 PHUB000503 PHUB001606	HMDB0031458 HMDB0030684 HMDB0037988	Plasma, urine	Common to many anthocyanin-rich foods; very low concentrations even after acute intake; very transient increase (< 2 h)	[79]
Cherry	6-Sulfatoxymelatonin	PHUB001884	HMDB0041815	Urine	Main metabolite of melatonin; common to all melatonin-rich foods. Also endogenous origin, affected by many factors (age, BMI, smoking…)	[84, 85]
Cherry	5-Hydroxyindoleacetic acid	PHUB001475	HMDB00763	Urine	Main metabolite of serotonin; common to all serotonin-containing foods such as banana, pineapple, and walnut; also endogenous origin	[88]
Plum	Anthocyanins: peonidin 3-glucoside, peonidin 3-rutoside, cyanidin monoglucuronide	PHUB001257; PHUB001296;	HMDB13689	Urine	Present in other berries, drupes, and red wine. Very low concentration and short half-life	[96]
Plum	Hippuric acid	PHUB001174	HMDB00714	Urine	Very common metabolite of polyphenols with many possible origins; not specific	[96]
Plum	Caffeic acid	PHUB000574	HMDB01964	Plasma, urine	Widely distributed in plant foods; not specific	[92]
Plum	5-Hydroxymethyl-2-furoic acid, (5-carboxylic acid-2-furoyl)glycine	PHUB001892 PHUB001894	–	Plasma, urine	Metabolites of 5-hydroxymethylfurfural (HMF), also present in coffee, dried fruit, fruit juices and honey	[93]
Cherry	Hydroxycinnamic acids: caffeic acid, ferulic acid, p-coumaric acid, vanillic acid	PHUB000574 PHUB000608 PHUB000316	HMDB0001964 HMDB0000954 HMDB0000484	Plasma	Widely distributed in plant foods; not specific	[97]

*Abbreviations*: *HMDB ID* human metabolome database identification number, http://www.hmdb.ca; PhytoHub ID: http://phytohub.eu/

#### Application of validation criteria

**Fig. 2:**
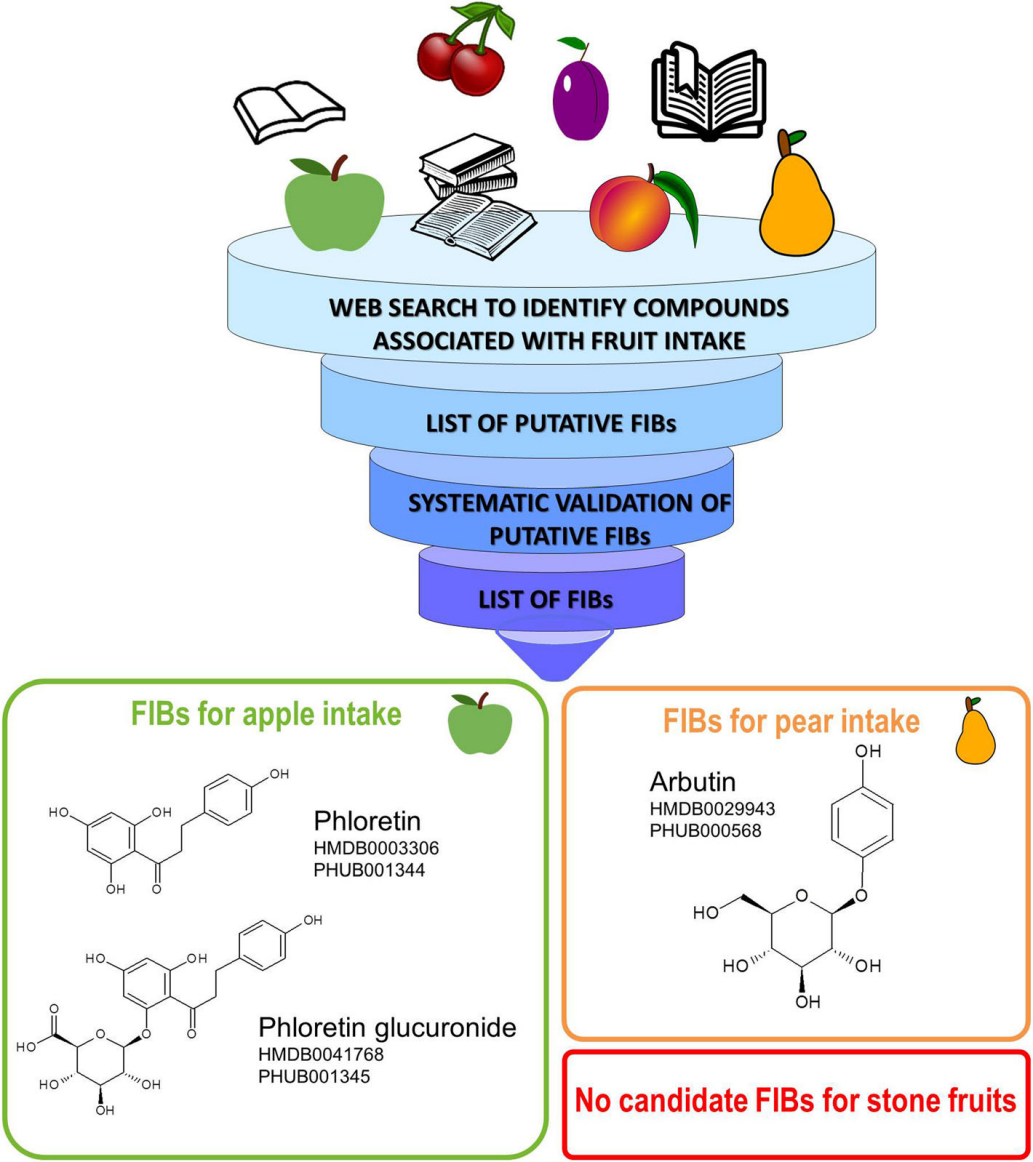
Scheme of literature evaluation process for pome fruits with BFIs: phloretin, phloretin glucuronide, and arbutin

According to Dragsted et al., a set of validation criteria was applied to the candidate BFIs reported in Table 1, in order to assess their current status of validation and to identify the missing information for a full validation of each of them [15]. The validation scheme is based on eight questions related to the analytical and biological aspects: Q1: Is the marker compound plausible as a specific BFI for the food or food group (chemical/biological plausibility)? Q2: Is there a dose-response relationship at relevant intake levels of the targeted food (quantitative aspect)? Q3: Is the biomarker kinetics described adequately to make a wise choice of sample type, frequency, and time window (time-response)? Q4: Has the marker been shown to be robust after intake of complex meals reflecting dietary habits of the targeted population (robustness)? Q5: Has the marker been shown to compare well with other markers or questionnaire data for the same food/food group (reliability)? Q6: Is the marker chemically and biologically stable during biospecimen collection and storage, making measurements reliable and feasible (stability)? Q7: Are analytical variability (CV%), accuracy, sensitivity, and specificity known as adequate for at least one reported analytical method (analytical performance)? Q8: Has the analysis been successfully reproduced in another laboratory (reproducibility)? Possible answers to each of mentioned questions were Yes, No or Unknown (Y/N/U), where “unknown” indicates lack of information in the literature. The current validation status of the candidate BFIs has been reported in Fig. 2.

### Results

#### Pome fruit

The literature search for biomarkers of apple intake resulted in 324 citations among which 46 articles were retained for full-text evaluation after screening of titles and abstracts, and 14 papers were finally selected after careful consideration of the inclusion criteria. Figure 1 shows a full flowchart with inclusion and exclusion criteria, and number of peer-reviewed papers in each part of evaluation. The main exclusion criteria were papers that discussed the effect of the intake of the different fruits on physiology, immunology, on diverse pathologies, or on drug metabolism, and animal or in vitro studies with unfitting design. Five observational studies, seven single-dose studies and two 4-week intervention studies reported associations between apple intake and plasma, urine or ileostomy fluid concentration of apple metabolites, which may represent candidate BFIs. A large majority of the selected articles described studies that aimed at quantifying polyphenol metabolites after apple intake and were motivated by an interest in the health effects of these bioactives. Two investigations used an untargeted metabolomics approach in cohort studies with the objective to identify the strongest associations between consumption of various food groups assessed by dietary questionnaires and metabolites identified in blood or urine metabolomes [17, 18]. A description of all studies reporting associations between apple intake and the most promising candidate BFIs for apple is given in Table 1. Four studies were not included in this table. Rago et al. [19] explored the effects of apple products consumption on the plasma metabolome but only reported changes in endogenous compounds, thus this article was not retained. Two studies, reported increased plasma concentration or urinary excretion of epicatechin after consumption of apple products [20, 21]. However, epicatechin has several other major dietary sources such as cocoa, tea, and other fruits (berries, apricots, grapes) [22–24] and is not specific enough to represent a candidate BFI for apple. In the fourth study, Pallister et al. [18] applied a metabolomics approach on 3500 fasting blood samples in a large twin cohort. Among the most significant associations observed were those between apple and pear intake and plasma threitol (0.033[0.003]; *P* = 1.69 × 10^−21^), indolepropionate (0.026[0.004]; *P* = 2.39 × 10^−9^), and 3-phenylpropionate (0.024[0.004]; *P* = 1.24 × 10^−8^). However, many confounders might explain these associations. Furthermore, the microbial metabolite 3-phenylpropionate is a non-specific degradation product of polyphenols such as naringin, ferulic acid, or proanthocyanidins [25]. Indolepropionate is a tryptophan metabolite produced by the gut microbiota, and no link with pome fruit consumption has been shown so far. Threitol is a sugar alcohol-like erythritol occurring naturally in many fruits and fermented foods. None of these three compounds was thus considered to be specific for apple consumption and the study was also excluded from Table 1. From the ten remaining studies, phloretin and phloretin glucuronide, most likely, the major metabolites of the apple dihydrochalcones phloridzin and phloretin 2′-*O*-xyloglucoside, were the only compounds consistently reported and finally retained for further evaluation as candidate BFIs for apple [26, 27]. An inventory of all the compounds observed after apple consumption but not retained as candidate BFIs is given in Table 2 with the main reasons for exclusion and original references. As the available data were quite limited, we also considered two investigations in rats fed apples during several weeks, as they used an untargeted mass spectrometry-based metabolomics approach likely to reveal new candidate biomarkers of intake that could be further studied in humans [28, 29]. Rago et al. [29] compared the plasma metabolomes of rats fed a control diet or the same diet supplemented with fresh apple slices. They found amino acids, carnitines, and lysophosphocholines as most discriminant metabolites, rather reflecting metabolic effects of apple than exposure to its components. Kristensen and coworkers [28] observed quinic acid, coumaric acid, epicatechin glucuronide, catechin glucuronide, methyl epicatechin, dihydroxyphenyl-valerolactone, hippuric acid, hydroxyhippuric acid, and homovanillic acid sulfate among the metabolites elevated in urine after apple intake. None of these can be considered as specific for apples. However, the authors shared a list of non-identified markers that increased significantly in all 24 animals after apple intake and not in the other group (7% apple pectins). These markers if found again discriminant in other metabolomics studies may deserve further identification work to provide specific biomarkers of apple intake, in complement to phloretin in urine.

Much less studies have been found for the other pome fruit pear and quince. Of the 338 papers retrieved from the primary literature search, 12 were eligible. After further assessment, only two papers were finally selected as relevant for pear BFIs (Table 1) while for quince no article was retained. Based on this very limited information, the only compounds that were retained as possible BFIs for pear were arbutin and hydroquinone sulfate. As for apple, some compounds listed in Table 2 were described in biofluids after pear intake but, for various reasons mostly related to a lack of specificity, were ruled out as candidate BFIs.

For the four candidate BFIs retained for apple and pear, validation criteria were examined and reported in Fig. 2 and further discussed below.

#### Stone fruit

A total of 433 references were screened for stone fruit, of which 11 original papers fulfilling the criteria were carefully examined, but none of them aimed at identifying biomarkers of intake. Of the retained references, seven described studies on cherry, including one single-dose study, four short-term (< 7 days), and two 4-week intervention studies. Six studies, all small size (involving 2 to 6 volunteers), focused on plum or prune. There was no data in the literature on potential BFIs for peach, nectarine, or apricot. No observational studies were published for stone fruit. Finally, no candidate BIF could be identified, even for cherry or plum from the literature survey. Table 3 lists all the metabolites reported to be associated with stone fruit intake but not retained as candidate BFIs, and the reasons for their exclusion are discussed below.

Of note, no biomarkers for the pome fruit and stone fruit as food groups emerged from the systematic search.

### Discussion

#### Apple biomarkers

Apple (*Malus domestica*) is the most consumed fruit in Europe [16], and one of most popular fruits worldwide [30, 31]. It represents the icon of healthy foods and has been suggested to exert protective effects towards CVD and other diseases [32–38]. Having a BFI for apple would be extremely useful to further document the effect of this healthy food, as well as for assessing dietary patterns of individuals, when used as part of a panel of BFIs covering major food groups. The nutrient composition of apples is well known [39]. Its most characteristic secondary metabolites are phloridzin (phloretin 2′-*O*-glucoside) and phloretin 2′-*O*-xyloglucoside, while other polyphenols such as epicatechin, proanthocyanidins, and chlorogenic acid are prevalent but common to many other food sources (e.g., other fruits such as berries, or green and black tea, coffee). Four observational studies have reported associations between phloretin and its derivatives and apple intake. Mennen et al. [40] searched correlations between the urine concentration of 13 polyphenols and the consumption of polyphenol-rich foods including apple, in the French SUVIMAX cohort study. They reported that apple consumption assessed with a 2-day dietary record was significantly correlated (*r* = 0.6, *P* < 0.0001) with phloretin concentration in morning spot urine and, to a lesser extent, with m-coumaric acid, isorhamnetin, kaempferol, and phloretin in 24-h urine samples [40]. Of these compounds, only phloretin can be considered as specific to apple. Kaempferol is present in many leaf vegetables, herbs, beans, and berries, m-coumaric acid is found in high content in olive, while isorhamnetin is a human metabolite of the widely distributed flavonol, quercetin [22–24]. A mean apple intake of 239.2 ± 95.0 g/day corresponded to a phloretin excretion of 0.73 ± 1.9 μmol/d and 0.42 ± 0.5 μmol/L in 24 h and spot urine, respectively [40]. Similar correlations were found between apple intake and phloretin urinary excretion in three observational studies by Krogholm et al. (79 volunteers) [41], Brantsæter et al. (119 volunteers) [42], and Edmands et al. (481 volunteers) [17]. Food intake was assessed with FFQ and 24-h recalls, while 24-h urine was analyzed with untargeted [17] or targeted approaches [41, 42]. Urinary excretion of phloretin (Krogholm et al.: *r* = 0.22, *p* < 0.01; Brantsæter et al., 2007: *r* = 0.25, *p* < 0.05) and phloretin glucuronide (AUC ROC curve = 75.8%, Edmands et al.) were found to be significantly correlated with “apple and pear” intake, but also with overall intake of fruit [17, 41, 42]. It can be speculated that among the population declaring a high consumption of fruit and fruit-based products, apples are particularly popular. Edmands et al. [17] also reported urine dihydroxyphenyl-γ-valerolactone sulfate and methyl(epi)catechin sulfate, two metabolites of epicatechin, to be correlated with apple and pear intake in addition to phloretin, but as discussed previously epicatechin is present in too many other food sources.

Some pharmacokinetics data are available for phloretin after consumption of apple or apple products in intervention studies. Phloretin was generally found at very low concentrations or below the limit of detection in plasma in the form of phloretin [43] or phloretin 2′-glucuronide [44, 45]. After consumption of 1 kg apples (Golden Delicious cultivar), the maximum phloretin concentration measured in men after enzymatic hydrolysis of plasma conjugates was 12.1 ± 6.6 nmol/L, with a Tmax in the range of 2.8–3.2 h [43]. Phloretin plasma Cmax and Tmax reported after cider and apple juice intake were in the same order of magnitude, although slightly higher than those after apple fruit intake: 73 ± 11 nmol/L and 0.6 ± 0.1 h after ingestion of 500 mL apple cider (nine subjects) [26] and 170 ± 90 nmol/L and 2.1 ± 0.5 h after intake of 1 L cloudy apple juice by five subjects [46]. Stracke and coworkers [43] reported that phloretin concentration was not increased in fasting plasma collected 24 h after the last intake of a 4-week supplementation period with 500 g apples per day in 43 men [43]. These data suggest that the half-life of phloretin is too short to allow accumulation in plasma. Furthermore, the maximum concentration reached in plasma after high intake of apple is very low (nmol/L range) suggesting that phloretin would be difficult to quantify for low to moderate apple intakes. The study by Rago et al. [19] is consistent with these data. Neither phloretin nor its metabolites were found among the discriminating markers in fasting plasma metabolomes of 24 volunteers after 4 weeks of supplementation with whole apples (550 g/day), clear and cloudy apple juices (500 ml/day), or dried apple pomace (22 g/day) compared to a non-supplemented diet [19].

Phloretin urinary excretion may be more useful to reflect the intake of apple and apple products. Phloretin is exclusively present in urine in conjugated forms, with a large predominance of phloretin-2′-*O*-glucuronide [26, 46]. After consumption of a single dose of 1 L cloudy apple juice, 0.54 μmol phloretin were measured in hydrolyzed 24 h urine, with 87% excreted in the 0–12-h period [46]. Compared to cloudy juice, phloretin excretion was shown higher after cider intake. After a 500 ml apple cider dose, Marks et al. [26], reported a phloretin excretion of 2.3 μmol in 24 h, with 98% of the dose excreted in the 0–8-h period in nine volunteers. Similarly, the urinary excretion of phloretin reached 3.8 ± 1.0 μmol in 24 h after 1.1 L apple cider [44]. Auclair et al. showed a dose-response relationship in urinary excretion of phloretin, measured after urine hydrolysis, in 30 subjects supplemented for 4 weeks in a cross-over design study with 40 g/d lyophilized apples of two varieties differing in their polyphenol content [47]. Phloretin concentrations in 24 h urine were 0.78 ± 1.17 nmol/mg and 0.52 ± 0.68 nmol/mg creatinine after the high and low polyphenol apples, respectively, which precisely reflected the 1.5 ratio in phloretin content of the two apple varieties. A dose-response relationship was also reported by Saenger et al. [48] who involved 30 healthy volunteers in an short-term parallel study with three doses of apples (200, 400, and 790 g) consumed in a context of a freely chosen background diet. Despite a high inter-individual variation and a low number of subjects, the urine concentration of phloretin quantified after urine hydrolysis was significantly different after the low and medium dose and after the high dose [48]. The kinetics study showed that the phloretin was rapidly excreted, with a maximum at 3 ± 1 h, and that 24 h after the apple intake the phloretin increase was no longer detectable [48].

The limited number of observational and human intervention studies available suggests that urinary excretion of phloretin and phloretin 2′-glucuronide are the most promising candidate biomarkers of recent apple intake [17, 41, 42]. According to an extensive search in the literature and online databases, no other significant dietary sources of phloretin glycosides have been reported so far. Strawberry can only be considered as a minor source of phloridzin, even in case of high consumption due to its very low content [49]. The peel of immature kumquat fruit contains a high amount of 3′,5′-Di-*C*-β-glucopyranosylphloretin (up to 2 g per 100 g dry peel); however, this fruit is barely consumed in most countries, and therefore, the probability that it becomes a confounding factor is rather low [50, 51]. In addition, of hundreds papers analyzing the composition to tomato, only four reported a minor content of the di-C-hexoside 3′,5′-Di- *C*-β-glucopyranosylphloretin in some varieties, and identification of the compound has never been confirmed by analysis of an authentic standard [52–55]. To our knowledge, the presence of phloretin or phloretin glucuronide was not detected so far in human or animal biofluids after tomato intake. Furthermore, C-glycosides are much less easily hydrolyzed than O-glycosides [56]; thus, it seems unlikely that tomato could also constitute a confounder for phloretin as a biomarker of apple intake.

Phloretin and its glucuronide are also associated with the intake of apple cider [26] and possibly other apple-based foods such as applesauce and juice. Apple processing for juice production has a major impact on phloridizin and related compounds. The dihydrochalcones are mostly associated with the solid parts of the fruit (peel and core zone) [57] and are poorly extracted into the juice. They are prone to oxidation, especially during the step of pulp enzyming when pectolytic enzymes are added to increase the juice extraction yield [58]. Only 10–20% of the original dihydrochalcone content of the fruit is recovered in the apple juice [58, 59]. In addition, the importance of apple varieties will have to be assessed, as the content in phloretin glycosides may vary significantly. The concentration of phloridzin, the main apple dihydrochalcone, has been recently screened among 247 apple genotypes and has been to be extremely variable within the *Malus* species, but relatively stable within the 150 cultivated apple analyzed (*Malus x domestica*) [60]. With the median concentration being 35.67 μg/g and with 75% of the 150 samples investigated within 64.8 μg/g, these data are in good agreement also with the total content on dihydrochalcones reported for the varieties most widely cultivated in Europe [60]. Therefore, we can assume that the concentration of apple dihydrochalcones is expected to have a limited variability within the cultivated apples, with the exception of the Renetta group (Renetta grigia, Renetta Canada, and Renetta Canada bianca), having a much higher concentration, up to a maximum of 310.6 μg/g [61]. Similarly cider apple varieties are about four times more concentrated than fresh consumed varieties [59], which explains the higher urinary excretion of phloretin observed after cider intake compared to apple juice intake [26, 44, 46]. But the latter, contrary to the Renetta group, are usually not considered for fresh consumption. To be taken into consideration as well is the fact that phloridzin is more concentrated in the skin, and thus, the mode of apple consumption should be registered since it may affect the relationship between the apple intake and the urinary excretion of phloretin glucuronide [62].

Only little information on the bioavailability of apple dihydrochalcones is available, obtained with low-dimension studies (*n* = 6–30) reported in this review, and possible inter-individual variation in the absorption and metabolism of these compounds will have to be further evaluated. Phloretin and phloretin glucuronide, because of their short half-life, belong to the short-term biomarkers category and repeated measurement will be necessary to assess the long-term consumption of apple. For the same reason, measurement in morning spot urine rather than in 24 h urine may less accurately reflect the apple intake from the previous day. Methods based on UPLC-MS/MS are available to quantify phloretin in human biofluids [63]. As phloretin 2′-glucuronide is largely predominant in urine, either the glucuronide or the aglycone after urine hydrolysis may be quantified. However, only the aglycone is commercially available as a standard.

To conclude, the urinary excretion of phloretin glucuronide or of phloretin measured after sample hydrolysis can be considered as the most promising specific biomarker of apple intake. The validation level of these candidate BFIs for apple is summarized in Fig. 2 which shows the need for more validation studies. The time window for urine collection and the dose-response relationships should notably be further documented. The standardization of a method of analysis will have to be carried out, which will include the conditions of the glucuronide hydrolysis. As the phloretin precursors, phoridzin and phloretin 2′-*O*-xyloglucoside, are expected to be present in all the apple products, the question arises, as for many other fruits, of the differentiation of the consumption of apple as a fruit or as juice or cider. This question is important as the categorization in food groups differs for apple and apple products in epidemiological studies and their health effects are likely to be divergent.

#### Pear biomarkers

Pear is a one of the oldest fruits cultivated in temperate regions, and many cultivars are now available. According to the comprehensive European food consumption database, pear is the second most eaten pome fruit after apple among the adult population, with a consumption ranging from 23 to 108 g/day [16]. Pear is mainly consumed fresh, but also canned and as juice. The available knowledge of the composition of pear, as well as the two studies performed by Nieman et al. [64] and Deisinger et al. [65] (Table 1), suggest that arbutin (hydroquinone-β-d-glucopyranoside) may be considered as a candidate biomarker of intake. Arbutin, along with other less specific phytochemicals such as oleanolic acid, ursolic acid, chlorogenic acid, rutin, and epicatechin, has been consistently detected in different cultivars of pear [66]. Interestingly, arbutin is commonly used to detect the adulteration of apple juices with pear in the food industry [67]. The concentration of this glycosylated hydroquinone ranges from 40 to 150 mg/l in pear juice and 6–113 mg/kg in fresh pear pulp [68]. Deisinger et al. reported that from a range of foods, pear was the richest source of arbutin with the skin reaching a concentration 40–60 times higher than the fruit’s flesh [65]. Fuentealba and coworkers have recently reported the presence of arbutin in walnuts of lighter color applying an untargeted metabolomics approach with a GC-MS method [69]. Oregano, marjoram, and wheat germ have also been reported to contain a high amount of arbutin; however, their lower level of consumption compared to pear does not make them major confounders for most populations [65, 70].

By applying an untargeted metabolomics approach, Nieman et al. [64] detected arbutin metabolites in human plasma samples after pear intake in a cross-over, randomized, controlled trial. In this study, the authors evaluated the effect of the intake of pear and banana on the physical endurance and recovery of 20 male athletes [64]. The results showed that arbutin and hydroquinone sulfate were the most discriminant metabolites of pear intake, along with various sugars, sugar acids (xylose, fructose, arabonate/xylonate, ribitol, mannitol/sorbitol, arabitol/xylitol), and some non-specific microbial metabolites of polyphenols (hippuric acid, 4-hydroxyhippurate, dihydroferulic acid, 4-allylphenol sulfate, vanillic alcohol sulfate, 3-(4-hydroxyphenyl)propionate). In the same study, eugenol sulfate and 2-isopropylmalate were found to increase after both pear and banana intake, opening their consideration as possible markers of total fruit intake [64]. Eugenol is an allyl alkoxybenzene present in a variety of food sources, such as spices, herbs, banana, and orange, and has been recovered in glucuronidated and sulfated form in healthy subjects [71]. 2-Isopropylmalate was identified as one of the key precursors for aldehydes and alcohols that contribute to the organoleptic properties of melon [72].

Deisinger et al. also reported the elevation of unidentified conjugated forms of hydroquinone in urine and plasma samples of four volunteers after the consumption of a hydroquinone and arbutin rich meal (784–1279 μg total hydroquinone) [65]. Two hours after the meal containing coffee, tea, wheat cereal, whole wheat bread, wheat germ, and Bosc pears, volunteers exhibited a fivefold elevation of hydroquinone in plasma (from 0.03 to 0.15 μg/g) while the urinary concentration of hydroquinone peaked 2–3 h after the meal (800 μg/h). The pharmacokinetics of arbutin in humans was studied in a randomized cross-over study in 16 healthy volunteers who consumed extracts of bearberry leaves, a non-dietary-rich source of arbutin [73]. About 65% of the 210 mg of the arbutin ingested was excreted in urine over a 24-h period, mostly in the first 4 h. Hydroquinone glucuronide was the major metabolite, accounting for 70% of the total arbutin metabolites while hydroquinone sulfate represented around 30%. Free hydroquinone was minor and barely detected [73]. The usefulness of the arbutin metabolite hydroquinone as a BFI of pear is challenged by the fact that human exposure to this metabolite is not limited to dietary sources. Environmental exposures include smoking, contact with cosmetic formulations such as nail polish and hair dyes, paint stabilizers, motor fuels, and photographic agents [74–76]. Deisinger and coworkers reported that after volunteers smoked four cigarettes in a 30-min period, plasma levels of hydroquinone increased to a lesser extent than after a hydroquinone-rich meal, from 0.015 to 0.030 μg/g within 10 min, and then decreased [65]. The urinary excretion of hydroquinone increased by a factor of 2.5 1 h after smoking [65].

**Fig. 3:**
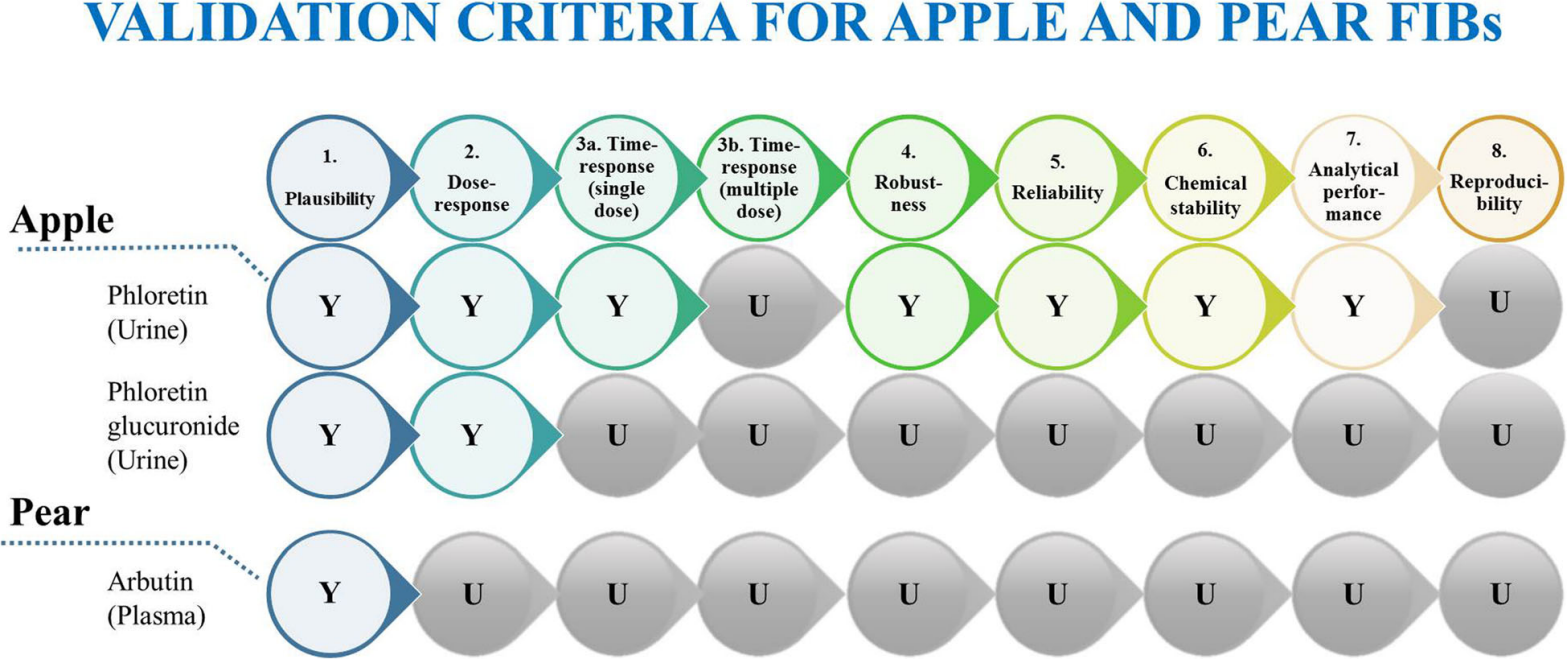
Overview of the validation process and its application process for candidate BFIs for pome fruits

In conclusion, although arbutin is used as a marker in the food industry to confirm the presence of pear in food products, data supporting its possible usefulness as BFI for pear are still scarce, as shown in Fig. 3. The findings of Nieman et al. [64] show that arbutin as such, not only its hydroquinone metabolites, is bioavailable after the intake of pear. The latter opens the possibility of using this compound as a marker of pear intake. In this regard, the analysis of urine after the intake of the fruit would offer an advantage over the study of plasma, as performed on both studies reviewed in this section. The analysis of urine generally allows a better insight of the metabolic footprint of food intake. Dose-response relationship and inter-individual variation after pear intake will have to be determined as these important validation criteria for BFIs have not been studied at all for arbutin excretion. The importance of confounding from dietary and environmental sources also need to be carefully assessed, in particular for use in large population studies.

#### Stone fruit biomarkers

Consumption of fresh cherry and other stone fruit is globally lower than that of pome fruit and is typically seasonal. For that reason, biomarkers of stone fruit intake may be mostly useful for monitoring compliance in studies investigating their health effects rather than for assessing the subjects’ adherence to a dietary pattern in the long term. The cherry fruit is a nutrient dense food containing a significant amount also of potentially bioactive food components, including chlorogenic acid isomers and anthocyanins [77]. As expected from the phytochemical composition of cherry, cyanidin glycosides were consistently found in plasma after cherry consumption in three intervention studies [78–80]. However, cyanidin glycosides are unlikely to serve as biomarkers of cherry intake for various reasons, including their chemical instability at neutral pH and their non-specificity to cherry (Table 3). Anthocyanins are also present in high content not only in a wide range of berries such as blackberry and blackcurrant, but also in grape, red onion, and red cabbage [81]. In fruit, anthocyanins usually exist as a complex mixture of conjugates with various sugars, hydroxycinnamates, and organic acids in proportions varying with the degree of fruit ripening. Furthermore, the total content of anthocyanins is increasing exponentially as the fruit ripens, which would challenge the dose-response relationship. In addition, cyanidin glycosides are detected in plasma for a very brief period of time, typically 1–2 h after intake and their concentration does not exceed a few dozens of nanomolar even after an acute intake of several portions of berries [82]. In a randomized, double-blinded, cross-over study on 12 healthy males, protocatechuic and vanillic acids were shown to increase in plasma following the intake of 30 or 60 mL of Montmorency tart cherry concentrate [83]. These phenolic acids are major metabolites of anthocyanins, such as cyanidin 3-glucoside, and are thus likely to derive from cherry anthocyanin metabolism, which means that again these compounds are not specific enough to cherry intake. The urinary excretion of the melatonin metabolite 6-sulfatoxymelatonin can increase (about 15–75%) after consumption of different varieties of cherry [84, 85]. However, cherry consumption cannot be considered as a major determinant of the 6-sulfatoxymelatonin plasma level, as it can originate from both an endogenous synthesis and other richer dietary sources of melatonin [86, 87]. The same conclusion applies for the urinary excretion of 5-hydroxyindoleacetic acid (5HIAA), which increased in 30 volunteers after consumption of cherries for 5 days [88]. 5HIAA is the main metabolite of serotonin. In humans, it can have an endogenous origin or derive from the consumption of serotonin-rich foods such as banana, pineapple, and walnut [89].

Other stone fruits for which a few relevant studies were obtained with the systematic literature search are plum and prune. Although their consumption is globally quite low, plum, especially when provided as a juice, is used as a functional food, mainly for its laxative effects [90]. Some people may therefore have a high intake of plum. As could be expected, some anthocyanins such as peonidin 3-glucoside, peonidin 3-rutoside, and the metabolites, cyanidin monoglucuronide and hippuric acid, were reported in urine, in a pilot study with plum juice [91], but as discussed above anthocyanins and their metabolites do not deserve further attention as candidate BFIs for any individual fruit. Caffeic acid was also detected in plasma and urine after a single intake of 100 g prunes, but this hydroxycinnamic acid is widely distributed in plant foods with coffee as a major source [92]. The only other metabolites reported so far as associated with plum consumption are metabolites of 5-hydroxymethylfurfural (HMF). In one study where plum was provided as juice, 5-hydroxymethyl-2-furoic acid (HMFA) and (5-carboxylic acid-2-furoyl) glycine were detected in plasma and urine [93]. HMF is formed from dehydration of fructose/glucose and Maillard reaction during heat-treatments like drying or cooking of sugar-containing foods. It has been identified in dried prunes and prune juice and in many thermally treated foods such as UHT milk, coffee, caramel, fruit juices, dried fruit, and honey and is also found in cigarette smoke [94]. Although HMF metabolites may have an interest when searching biomarkers of intake of processed foods, they are not specific enough to be further investigated as BFIs for plums.

In conclusion, almost everything remains to be done in the field of intake biomarkers for stone fruit. Every fruit has a specific phytochemical signature of hundreds of compounds, and the exploration of this complexity might reveal more specific candidate biomarkers among the phytochemicals. Many of the fruit phytochemicals have not been selected for targeted analyses so far since they were not associated with an already known biological activity. However, databases containing information on the phytochemical composition of foods, such as FooDB, PhytoHub, Dr. Duke’s Phytochemical and Ethnobotanical Databases, and CRC Dictionary of Food Compounds, show the incredible diversity of compounds in fruit, far beyond the most-studied polyphenols and carotenoids. In the particular case of stone fruit, these databases reveal specific phytochemicals, such as cerasinone (7-hydroxy-2′,4′,5-trimethoxyflavanone) and cerasin in sour cherry and ephedrannin A, syringetin 3-robinoside, and betuletol in apricot. After absorption and metabolism, these species-specific phytochemicals may lead to metabolites representing new candidate biomarkers of stone fruit intake. Untargeted metabolomics applied to intervention studies is of particular interest for discovering such specific metabolites in a data-driven approach, with a particular attention on trying to identify the expected metabolites of the specific phytochemicals from different species of stone fruit.

## General conclusion

The extensive literature search conducted under the well-defined criteria of the BFIRev protocol demonstrated the limited knowledge available so far regarding the possible biomarkers of intake for pome and stone fruit. Only urinary phloretin seems applicable for apple; however, more validation work is needed before its level can be translated into a value, or more likely a value range, of apple consumption. The high interest raised recently on the discovery of new biomarkers of food intake and the development of good practices for using metabolomics for that purpose will certainly lead to the publication of many new candidate biomarkers. The sharing of metabolomics data acquired in independent observational and intervention studies will allow the rapid examination of the specificity and other validation criteria of the newly discovered biomarkers.
